# Age differences in the treatment of lung cancer–a cohort study among 42,000 patients from Germany

**DOI:** 10.1007/s00432-024-06025-5

**Published:** 2024-11-15

**Authors:** Nikolaj Rischke, Josephine Kanbach, Ulrike Haug

**Affiliations:** 1 Department of Clinical Epidemiology, Leibniz Institute for Prevention Research and Epidemiology–BIPS, Achterstr. 30, 28359 Bremen, Germany; 2 Faculty of Human and Health Sciences, University of Bremen, Bremen, Germany

**Keywords:** Lung cancer, Anticancer therapy, Age differences, Real-world data

## Abstract

**Aims:**

We aimed to describe treatment of lung cancer patients in Germany based on health claims data, focusing particularly on differences by age.

**Materials and methods:**

Using the German Pharmacoepidemiological Research Database (GePaRD, ~ 20% of the German population) we identified lung cancer patients diagnosed in 2015–2018 based on a previously developed algorithm and followed them until death, end of continuous insurance or end of 2020. We described initial treatment patterns after diagnosis and survival, stratified among others by age.

**Results:**

We included 42,629 incident lung cancer patients (58% male). Surgery within three months after diagnosis was performed in 36%, 31%, 29% and 18% of patients aged < 50, 50–69, 70–79 and ≥ 80, respectively. Among patients without surgery, systemic therapy was administered in 77%, 72%, 54% and 25% of patients aged < 50, 50–69, 70–79 and ≥ 80, respectively. Monoclonal antibodies were administered in 15–30% of patients across age groups, and 4% to 15% received protein kinase inhibitors. Overall, 21% of patients remained untreated. In the age groups < 50, 50–69, 70–79 and ≥ 80, this proportions was 9%, 12%, 22% and 48%, respectively.

**Conclusion:**

In conclusion, our study provides a comprehensive overview of the therapy of lung cancer patients in Germany and quantitatively demonstrates the considerable differences between age groups. In terms of clinical cancer registration, the results are useful to estimate the completeness of data for the different types of treatment.

**Supplementary Information:**

The online version contains supplementary material available at 10.1007/s00432-024-06025-5.

## Introduction

Lung cancer is one of the leading causes of cancer morbidity and mortality worldwide (Thandra et al. 2021). In Germany, nearly 60,000 patients were diagnosed with lung cancer and 45,000 patients died of the disease in 2019; the absolute five-year survival was 15% in men and 20% in women (Krebs in Deutschland für 2019). Treatment options for lung cancer include chemotherapy, radiotherapy and surgery. In addition, several new targeted anticancer therapies have been developed in the last two decades (Lungenkrebs 2023).

To monitor the course of cancer patients and assess cancer care in the real-world setting, clinical cancer registries have been established in Germany, following the adoption of the so-called Cancer Screening and Registry Act in 2013. Although clinical cancer registries are now fully implemented, it is still unclear whether the information on therapy is complete. There are no established indicators in this regard, for example, which proportion of lung cancer patients is expected to receive chemotherapy. To establish such indicators, health claims data are a useful data source given that these therapies are reimbursed by health insurance providers. In addition to overall proportions, the indicators can also be determined stratified by age, which is important given the expected differences in treatment patters by age. For example, a study among breast cancer patients based on German health claims data showed that the proportion of patients receiving chemotherapy within four months of breast surgery was 63% for patients under 50, 46% for patients aged 50–69, 27% for patients aged 70–79 and only 4% for patients aged 80 or older (Heinig et al. 2022). This illustrates the importance of quantifying differences by age and taking them into account when estimating the completeness of treatment information.

For lung cancer, there is no up-to-date and comprehensive description of treatment patterns in Germany. Using a large German claims database, we therefore aimed to investigate treatment of lung cancer patients in Germany, focusing particularly on differences by age.

## Methods

### Data source

Our study was based on the German Pharmacoepidemiological Research Database (GePaRD) which contains health insurance claims data from four statutory health insurance (SHI) providers in Germany. The database currently includes information on approximately 25 million persons who have been insured with one of the participating SHI providers since 2004 or later. In addition to demographic data, GePaRD contains information on drug dispensations as well as outpatient (i.e., from general practitioners and specialists) and inpatient services and diagnoses. Per data year, there is information on approximately 20% of the general population and all geographical regions of Germany are represented.

Diagnoses in GePaRD are coded according to the International Classification of Diseases 10th revision, German Modification (ICD-10-GM). We used a previously developed algorithm to identify incident lung cancer cases in claims data. Incidence determined based on this algorithm was plausible when compared to cancer registry data. Information on stage at diagnosis is not available in claims data but was roughly estimated based on ICD codes indicating lymph node involvement or distant metastases as previously described (Oppelt et al. 2019).

### Study population and study design

We included patients with incident lung cancer diagnosed between 2015 and 2018. Patients with inconsistent or missing information on sex or birth year as well as patients not living in Germany were excluded. For included patients, we assigned the date of the first code for lung cancer as cohort entry and followed them until death, end of continuous health insurance or end of the study period (December 31st 2020), whichever occurred first.

### Assessment of lung *cancer* therapy

We assessed (I) if there was a lung cancer surgery within three months after cohort entry, (II) if those with surgery had a neoadjuvant therapy, (III) if there was a systemic anticancer therapy within six months after cohort entry and (IV) if there was a radiotherapy within eleven months after cohort entry. Systemic anticancer therapy was categorized into cytostatic drugs, monoclonal antibodies (including checkpoint inhibitors such as nivolumab) and protein kinase inhibitors.

### Data analysis

We described all included patients regarding baseline characteristics. We calculated the proportion of patients with surgery within three months after diagnosis and among those the proportion receiving neoadjuvant therapy, the proportion of patients with systemic anticancer therapy initiated within six months after diagnosis and the proportion of patients receiving radiotherapy within eleven months after diagnosis. We described overall survival after diagnosis using Kaplan–Meier analyses.

We stratified the analysis by age at cohort entry (< 50 years, 50–69 years, 70–79 years, 80 + years) and stage at cohort entry (advanced including metastatic stage vs. non-advanced). For all analyses, we used the software SAS version 9.4 (SAS Institute Inc., Cary, NC).

## Results

### Baseline characteristics

Overall, we included 42,629 lung cancer patients (57.6% male). The mean age at diagnosis was 69.2 years in men and 68.3 years in women. The distribution of included lung cancer patients by age group and sex is shown in Supplementary Table 1. The proportion assigned to the category “advanced stage at diagnosis” (including metastatic stage) was 70.7% in women and 71.1% in men. Among those with advanced stage, 70% had distant metastases at the time of diagnosis. The most frequent locations of distant metastases were “bone/bone marrow”, followed by “brain” in women and “liver” in men (Table 1).

**Table 1:** Baseline characteristics of included lung cancer patients

	Women	Men
N (%)	18.081 (100)	24.548 (100)
Year of incident diagnosis, n (%)		
2015	4.274 (23.6)	6.119 (24.9)
2016	4.553 (25.2)	6.089 (24.8)
2017	4.672 (25.8)	6.277 (25.6)
2018	4.582 (25.3)	6.063 (24.7)
Age at diagnosis [years]		
Mean ± standard deviation	68.3 ± 10.7	69.2 ± 10.4
Age groups, n (%)		
< 50	669 (3.7)	712 (2.9)
50–69	8.967 (49.6)	11.489 (46.8)
70–79	5.817 (32.2)	8.332 (33.9)
80 +	2.628 (14.5)	4.015 (16.4)
Stage at diagnosis, n (%)		
Non-advanced	5.292 (29.3)	7.092 (28.9)
Advanced	12.789 (70.7)	17.456 (71.1)
Among those, cases withdistant metastases^a^, n (% of advanced)	9.218 (72.1)	12.183 (69.8)
Bone/bone marrow	3.349 (26.2)	4.746 (27.2)
Brain	2.962 (23.2)	3.540 (20.3)
Liver	1.660 (13.0)	2.140 (12.3)
Adrenal gland	2.668 (20.9)	3.695 (21.2)
Mediastinum/Pleura	2.010 (15.7)	2.559 (14.7)

^a^According to codes recorded in the quarter of incident lung cancer diagnosis or the following quarter

### Treatment patterns

**Fig. 1:**
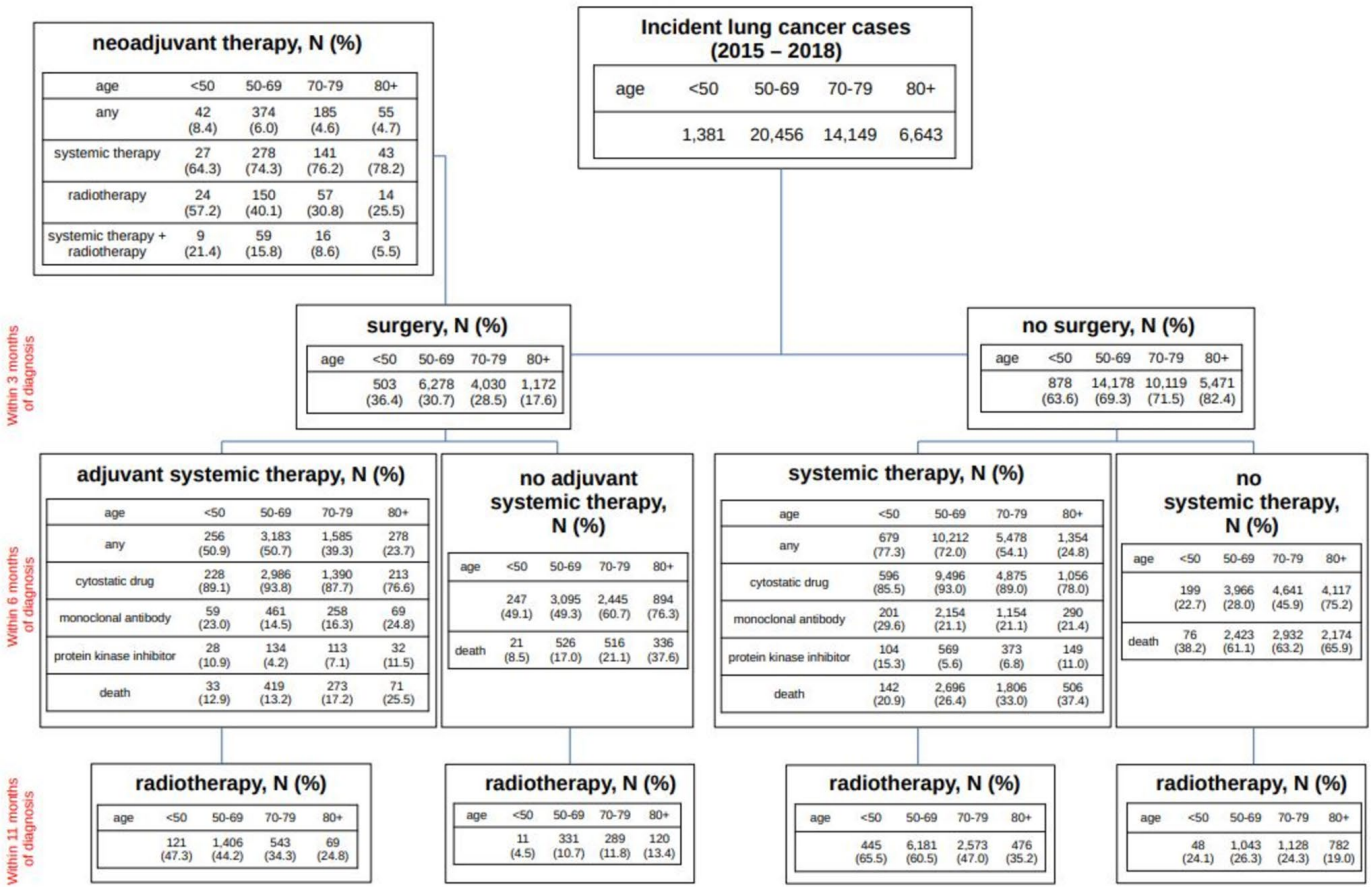
Treatment of included lung cancer patients stratified by age group. The time frames considered for the different types of treatment are shown on the left hand-side (in red). In addition, the number of deaths occurring within six months after diagnosis is shown

Figure 1 shows the treatment patterns within eleven months after diagnosis, stratified by age group. Surgery within three months after diagnosis was performed in 36%, 31%, 29% and 18% of patients aged < 50, 50–69, 70–79 and ≥ 80, respectively. Of those, 5%–8% received neoadjuvant therapy. Systemic therapy after surgery was administered in 24%–51% of patients. The proportion of patients with surgery who received monoclonal antibodies ranged from 15 to 25%. In patients aged 50–79 years, the proportion receiving protein kinase inhibitors was below 10%, overall; in younger and older patients, it was above 10%. 25–47% of patients who received adjuvant systemic therapy also received radiotherapy. In patients without adjuvant systemic therapy, these proportions were 5–13%.

Among patients without surgery, systemic therapy was administered in 77%, 72%, 54% and 25% of patients aged < 50, 50–69, 70–79 and ≥ 80, respectively. Monoclonal antibodies were used in 21% of patients 50 or older. In patients aged 50–79 years, the proportion receiving protein kinase inhibitors was below 7%, in younger and older patients, it was above 11%. 35%–66% of patients who received systemic therapy also received radiotherapy. In patients without systemic therapy, these proportions were 19%–26%.

Supplementary Figs. 1 and 2 show the results from Fig. 1, distinguishing between patients with advanced vs. non-advanced stage at diagnosis.

Overall, 8,897 (20.9%) patients received no cancer-directed therapy. The mean age in this subgroup was 75 years. 7968 (89.6%) of the untreated patients had died by the end of the observation period. In the age groups < 50, 50–69, 70–79 and 80 or older the proportions of untreated patients were 8.8%, 12.1%, 22.1% and 47.7%, respectively.

### Overall survival

**Fig. 2:**
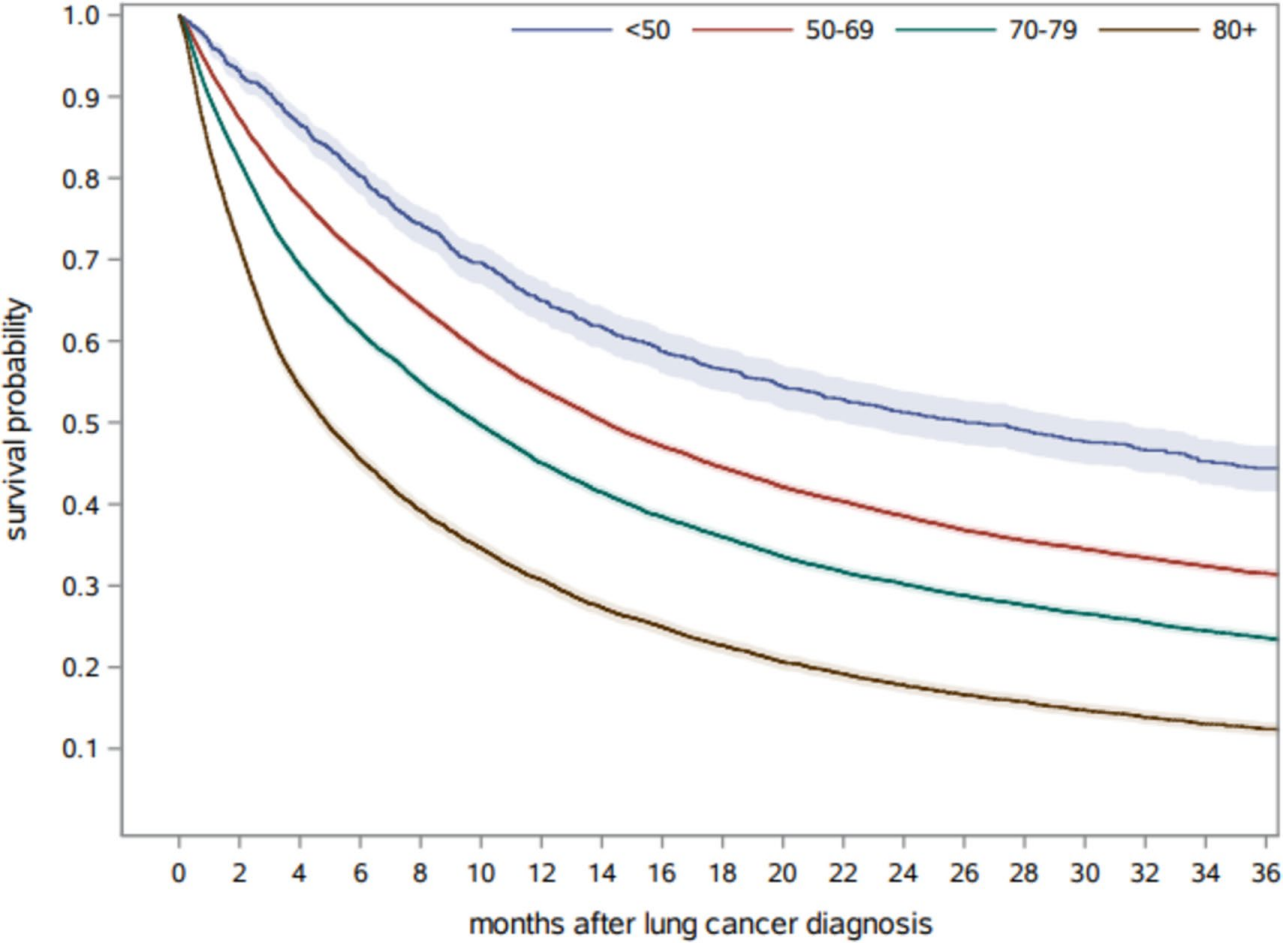
Overall survival of included lung cancer patients stratified by age group

Of the 42,629 lung cancer patients, 31,825 (74.7%) died during follow-up; 36.4% died within six months. Figure 2 shows overall survival stratified by age groups. The median overall survival was 26 months, 14 months, 10 months and 5 months for patients aged < 50, 50–69, 70–79 and ≥ 80, respectively. Survival stratified by sex is shown in Supplementary Fig. 3. With 52%, women had a higher 12-month survival probability compared to 45% in men.

## Discussion

Our study provides a comprehensive and up-to-date overview of real-world treatment patterns among lung cancer patients in Germany. Including more than 42,000 patients diagnosed in 2015–2018, we quantified proportions of different treatments (systemic therapy, surgery, radiotherapy) by age and found marked differences. For example, surgery was conducted in about 30% of patients aged 50–79 vs. 18% of patients aged 80 or older. In patients without surgery, 72% of patients aged 50–69 were treated with systemic anticancer therapy vs. 54% of patients aged 70–79 and 25% of patients aged 80 or older.

To our knowledge, there is only one study from Germany investigating treatment of lung cancer patients according to age (Walter et al. 2019). The authors of this study, which included 13,283 patients, concluded that the likelihood of receiving tumor-directed treatment was lower in patients above 65 as compared to younger patients. However, the included patients were diagnosed in 2009. Since then, treatment options were extended, e.g., due to the rapidly evolving landscape of new therapeutics, especially monoclonal antibodies and tyrosine kinase inhibitors. Furthermore, follow-up was limited to six months in this study.

Studies investigating age-related patterns in the use of tumor-directed therapy from other countries that are comparable to our study (i.e., focus on the whole group of lung cancer patients) are also scarce and only included cancer patients diagnosed before 2012 (Nilssen et al. 2016; Costa et al. 2017). In a study from Norway including 24,324 lung cancer patients diagnosed between 2002 and 2011 the proportion receiving surgery was 23% in patients aged 50–69 years, 18% in patients aged 70–79 years and 5% in patients aged 80 years or older (Nilssen et al. 2016). A study from Brazil including 40,403 lung cancer patients diagnosed between 2000 and 2011 compared treatment in patients < 70 vs. ≥ 70 years. While the proportion receiving surgery was rather similar in both age groups and in those < 70 years lower compared to our study (18% vs. 16%), the proportion receiving systemic anticancer therapy showed an age gradient (62% vs. 49%) (Costa et al. 2017).

In our study, 21% of included lung cancer patients did not receive any cancer-directed treatment. This proportion increased with age: it was 12% in patients aged 50–69 years, 22% in patients 70–79 years and 48% in patients aged 80 years or older. Also in this regard, there are only a few comparative studies that focus on the entire group of lung cancer patients. A study from Australia including 1116 lung cancer patients with a mean age of 72 years diagnosed between 2006 and 2013 as well as a study from Korea including 2,148 lung cancer patients diagnosed between 2009 and 2014 both reported a proportion of 28% untreated patients (Ngo et al. 2022; Choi et al. 2019). When weighing up the potential benefits and harms in older patients, the higher prevalence of comorbidities and poorer performance status as compared to younger patients certainly influence treatment decisions. On the one hand, the physician may assess the risks to be greater than the benefits and therefore advise against treatment. On the other hand, the patient may also decide against treatment for other reasons. A systematic review that summarized studies describing factors that led older adults to accept or decline cancer treatment found considerable variation in the underlying reasons, but the most consistent determinant was physician recommendation. However, the review also emphasized the need for further studies that are based on large, representative samples and examine decision-making taking into account health literacy and comorbidity (Puts et al. 2015).

From the perspective of clinical cancer registration, knowledge on the extent of the age gradient for the different types of treatment as well as the proportion of untreated patients is of high importance. For epidemiological cancer registration, there are various established methods for estimating completeness, i.e. the extent to which all of the incident cancers occurring in the population are included in the registry (Parkin and Bray 2009). To estimate the completeness of treatment information for incident cancers, however, quality indicators are lacking. Without reference values, clinical cancer registration lacks guidance as to whether the data are sufficiently complete to describe cancer care or whether further measures need to be taken due to a lack of completeness. Based on the results of our study, the expected proportions can roughly be estimated for the different types of treatment. It is also possible to take into account the age distribution of the incident patients recorded in the respective registry and combine it with our age-specific values, which allows the expected proportions to be estimated even more accurately. Even though the accuracy of such quality indicators could certainly be further improved, the analyses presented here based on health claims data can be useful as a first pragmatic approach to assessing the completeness of treatment data from clinical cancer registration.

Our study had strengths and limitations. Strength include the large sample size, the sophisticated algorithm used to ensure the valid identification of lung cancer patients in claims data, the continuous follow-up as well as the completeness of information regarding therapy in the inpatient and outpatient setting. Completeness in claims data does not depend on active reporting by physicians as it is the case in cancer registry data, and there is no bias due to non-responder as it is the case in survey data. Unlike cancer registry data, however, claims data lack detailed information on stage at diagnosis and there is no information on histology. Some advanced stages may have been misclassified as non-advanced if the patients died shortly after diagnosis without any treatment that would have prompted the coding of affected lymph nodes or distant metastases. These limitations are inherent to German health claims data but in the absence of other data sources, health claims data are still useful for the purposes described above. A record linkage of cancer registry and health claims data combining the advantages of both databases would be ideal and there are currently legislative prospects in this respect, but it will be a while before this is implemented.

## Conclusion

Our study provides a comprehensive overview of the therapy of lung cancer patients in Germany and quantitatively demonstrates the considerable differences between age groups. In terms of clinical cancer registration, the results are useful as they provide reference values for estimating the completeness of data for the different types of treatment. Thus, the results support clinical cancer registries in optimizing their data quality. Only with good data quality will it be possible for clinical cancer registries to fulfil their purpose, i.e. monitoring and optimizing the quality of cancer care.

## Conflict of interest

sThe authors declare no competing interests.

## Ethical approval

Informed consent for studies based on claims data is required by law unless obtaining consent appears unacceptable and would bias results, which was the case in this study. According to the Ethics Committee of the University of Bremen studies based on GePaRD are exempt from institutional review board review.

## Supplementary Information

Below is the link to the electronic supplementary material.

Supplementary file1 (DOCX 327 KB)

## Data Availability

No datasets were generated or analysed during the current study.
