# Supplementary material for: Age differences in the treatment of lung cancer–a cohort study among 42,000 patients from Germany
Source: J Cancer Res Clin Oncol. 2024 Nov 15;150(11):503. doi: 10.1007/s00432-024-06025-5 (PMC11568014; doi:10.1007/s00432-024-06025-5)

**Article title: Age differences in the treatment of lung cancer – a cohort study among 42,000 patients from Germany**

Nikolaj Rischke ^1^, Josephine Kanbach ^1^, Ulrike Haug ^1,2^

1. Department of Clinical Epidemiology, Leibniz Institute for Prevention Research and Epidemiology – BIPS, Bremen, Germany.

2 Faculty of Human and Health Sciences, University of Bremen, Germany.

***Author for correspondence:**

Prof. Dr. Ulrike Haug
Department of Clinical Epidemiology
Leibniz Institute for Prevention Research and Epidemiology – BIPS
Achterstr. 30
28359 Bremen, Germany
Tel.: +49-(0)421-218-56862
E-mail: [haug@leibniz-bips.de](mailto:haug@leibniz-bips.de)

**Supplementary Table 1: Proportion of lung cancer patients classified as advanced at diagnosis stratified by sex and age group**

|  | All | | Women | | Men | |
| --- | --- | --- | --- | --- | --- | --- |
|  | Total | advanced* | Total | advanced* | Total | advanced* |
| Total | 42,629 | 30,245 (70.9) | 18,081 | 12,789  (70.7) | 24,548 | 17,456  (71.1) |
| <50 | 1,381 | 1,035 (74.9) | 669 | 488  (72.9) | 712 | 547  (76.8) |
| 50-69 | 20,456 | 15,262 (74.6) | 8,967 | 6,634  (74.0) | 11,489 | 8,628  (75.1) |
| 70-79 | 14,149 | 9,697 (68.5) | 5,817 | 3,995  (68.7) | 8,332 | 5,702  (68.4) |
| 80+ | 6,643 | 4,251 (64.0) | 2,628 | 1,672  (63.6) | 4,015 | 2,579  (64.2) |

* Please note that in the interpretation of the proportion of stage by age, it has to be considered that a relatively high proportion of older patients does not receive any treatment. We assume that this also affects the coding of affected lymph nodes and distant metastasis, i.e. if there is no treatment, it may also be less likely that these ICD codes are in the claims data. The lower proportion of advanced stage in patients aged 70 years or older as compared to younger patients should therefore be interpreted with caution.

**Supplementary Figure 1**: Treatment of included lung cancer patients diagnosed in a non-advanced stage; stratified by age group. The time frames considered for the different types of treatment are shown on the left hand-side (in red). In addition, the number of deaths occurring within six months after diagnosis is shown.


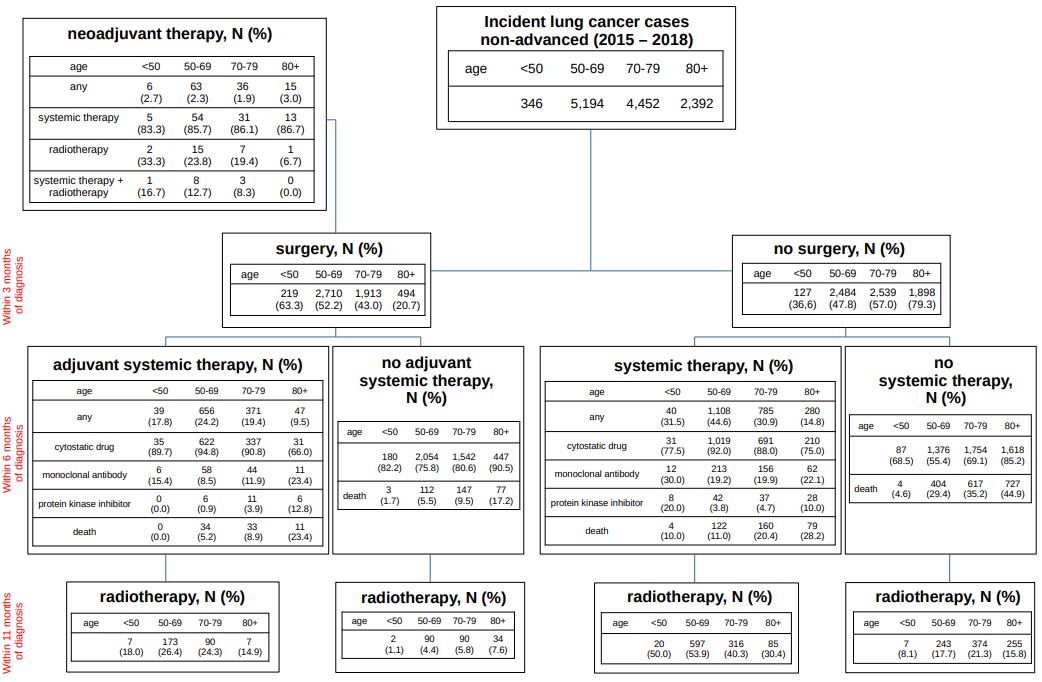


**Supplementary Figure 2**: Treatment of included lung cancer patients diagnosed in a advanced stage; stratified by age group. The time frames considered for the different types of treatment are shown on the left hand-side (in red). In addition, the number of deaths occurring within six months after diagnosis is shown.

**
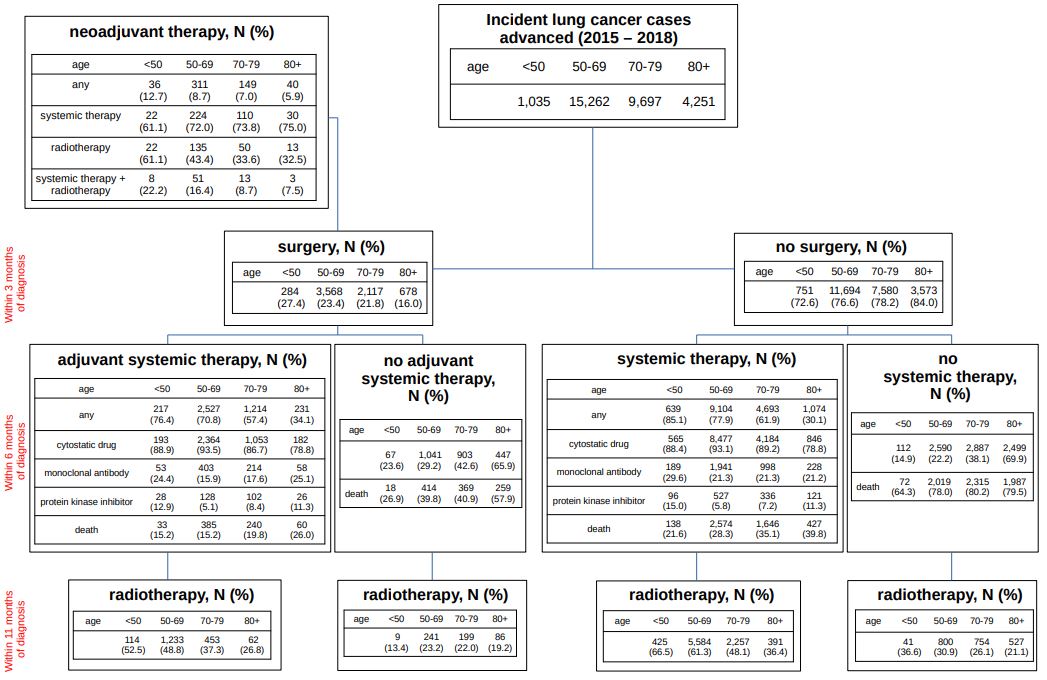
**

**Supplementary Figure 3**: Overall survival of included lung cancer patients stratified by sex.


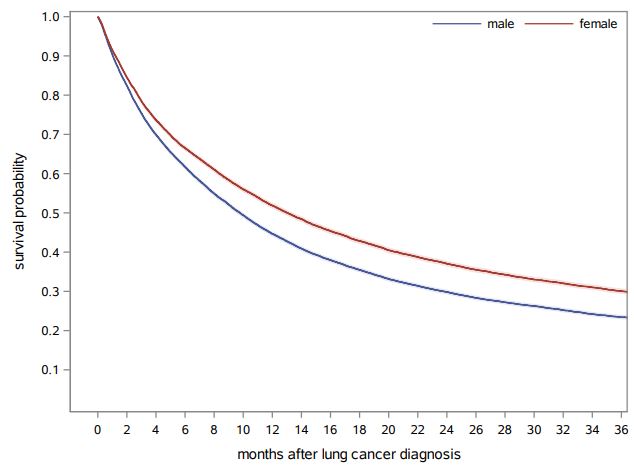

Supplement: Supplementary file 1 — Supplementary file1 (DOCX 327 KB) [file 432_2024_6025_MOESM1_ESM.docx]
